# Measuring Sexual Behavior Stigma to Inform Effective HIV Prevention and Treatment Programs for Key Populations

**DOI:** 10.2196/publichealth.7334

**Published:** 2017-04-26

**Authors:** Shauna Stahlman, James R Hargreaves, Laurel Sprague, Anne L Stangl, Stefan D Baral

**Affiliations:** ^1^ Center for Public Health and Human Rights Department of Epidemiology Johns Hopkins University Baltimore, MD United States; ^2^ Department of Social and Environmental Health Research London School of Hygiene & Tropical Medicine London United Kingdom; ^3^ HIV Justice Network Detroit, MI United States; ^4^ Irvin D Reid Honors College Wayne State University Detroit, MI United States; ^5^ Department of Global Health Youth and Development International Center for Research on Women Washington, DC United States

**Keywords:** stigmatization, social stigma, HIV, male homosexuality, sex workers

## Abstract

**Background:**

The levels of coverage of human immunodeficiency virus (HIV) treatment and prevention services needed to change the trajectory of the HIV epidemic among key populations, including gay men and other men who have sex with men (MSM) and sex workers, have consistently been shown to be limited by stigma.

**Objective:**

The aim of this study was to propose an agenda for the goals and approaches of a sexual behavior stigma surveillance effort for key populations, with a focus on collecting surveillance data from 4 groups: (1) members of key population groups themselves (regardless of HIV status), (2) people living with HIV (PLHIV) who are also members of key populations, (3) members of nonkey populations, and (4) health workers.

**Methods:**

We discuss strengths and weaknesses of measuring multiple different types of stigma including perceived, anticipated, experienced, perpetrated, internalized, and intersecting stigma as measured among key populations themselves, as well as attitudes or beliefs about key populations as measured among other groups.

**Results:**

With the increasing recognition of the importance of stigma, consistent and validated stigma metrics for key populations are needed to monitor trends and guide immediate action. Evidence-based stigma interventions may ultimately be the key to overcoming the barriers to coverage and retention in life-saving antiretroviral-based HIV prevention and treatment programs for key populations.

**Conclusions:**

Moving forward necessitates the integration of validated stigma scales in routine HIV surveillance efforts, as well as HIV epidemiologic and intervention studies focused on key populations, as a means of tracking progress toward a more efficient and impactful HIV response.

## Introduction

### Sexual Behavior Stigma

As defined by Joint United Nations Program on human immunodeficiency virus infection and acquired immune deficiency syndrome (HIV/AIDS; UNAIDS), key populations are groups of people who are more likely to acquire or transmit HIV, and whose engagement is necessary for a successful HIV response [1]. Key populations include people living with HIV (PLHIV), men who have sex with men (MSM), transgender people, people who inject drugs, sex workers and their clients, and others. However, in this paper, we will focus primarily on MSM and sex workers and the sexual behavior-related stigma affecting these groups. Incidence and prevalence data indicate high and sustained HIV epidemics among MSM and sex workers across all income levels [2-18], as well as HIV care cascade outcomes that are far below the 90-90-90 target set by UNAIDS for 2020 [19].

Stigma toward key populations has been linked with adverse HIV-related outcomes that prevent reaching 90-90-90 testing and treatment targets. For example, among MSM, experience of stigma has been associated with reduced rates of HIV testing, increased fear and avoidance of seeking health care, and having condomless anal sex [20-26]. Interaction with health workers, who have not been properly trained in MSM clinical and cultural competency, may result in MSM avoiding HIV prevention or treatment services. In addition, reduced utilization of health and HIV services by MSM, due to enacted or perceived discrimination, may limit knowledge of the risks of unprotected anal intercourse and opportunities for access to prevention services [27,28]. These associations with HIV risk-related outcomes are likely amplified through the intersection of other types of stigma including stigma due to HIV status, race or ethnicity, or gender identity, as well as membership in multiple key population groups (eg, MSM sex workers) [29-31]. Similar patterns have been reported for female sex workers [32-34]. Consequently, the World Health Organization, UNAIDS, and the White House National HIV and AIDS Strategy recognize the important role that stigma plays in contributing to these negative outcomes and stress the need to reduce stigma affecting key populations [35-37]. Better understanding of the role that stigma plays in shaping HIV-related outcomes, tracking trends in stigma, and understanding the impact of interventions and policy changes on stigma all require accurate approaches to stigma surveillance and measurement.

Stigma is defined here as a social process that labels some people within a larger community as less valuable than others based on certain characteristics [38,39]. Several types of stigma have been characterized. Perceived stigma, also referred to as felt [40], or felt-normative [41] stigma, refers to the belief that individuals or societies treat people differently due to a stigmatized characteristic. Anticipated stigma is the fear or expectation of stigma or discrimination [42], whereas enacted or experienced stigma is the explicit experience of poor treatment on the basis of specific characteristics [43,44]. Internalized or self-stigma, which refers to the acceptance of one’s lesser status within a society, manifests in low self-esteem, self-isolation, and social withdrawal [45,46]. More recently, concern has also been raised about the severity and potential deadliness of internalized stigma in the form of internalized homophobia and violence [47]. Finally, intersecting stigma occurs when multiple forms of stigma interact within one individual or group, such as those related to being gay and living with HIV [48,49]. Stigma can result in discrimination, which has been defined as being treated differently based on an ascribed or perceived trait [50].

Stigma and discrimination affecting key populations can be caused by unequal access to social, economic, and political power that allows for separation, labeling, and stereotyping of groups [39]. In a social process, HIV is presented as preventable, with “immoral” behaviors causing the infections. Key populations are thus blamed for their own HIV infection, which can result in even further disadvantage [51]. Given the high levels of stigma affecting key populations, the consistent associations between stigma and adverse health outcomes, and the demonstrated ability to intervene on stigma through structural interventions or reduction of internalized stigma [52-56], sexual behavior stigma should be a priority to implement in surveillance efforts. Yet despite significant rhetoric from multiple HIV and health organizations highlighting the need to measure and address stigma, we found in a systematic review that most epidemiological and surveillance studies among MSM and female sex workers continue to focus on individual-level risks including numbers of partners and condomless anal sex, which have been well-established as risk factors of HIV infection [3,12,34]. Another systematic review of 549 English, French, and Spanish peer-reviewed articles that measured sex worker- or MSM-associated stigma from 2004 to 2014 identified a dearth of validated stigma metrics toward key populations in low- and middle-income settings (particularly in Africa and in the Middle East), a disproportionate lack of data on sex workers, and lack of studies utilizing mixed methods [57].

### Outline for This Study

In this paper, we outline a vision for appropriate surveillance of sexual behavior stigma, defined as stigma that is anticipated, perceived, or experienced as a result of one’s sexual practices [58,59]. Conceptually, sexual behavior stigma has considerable overlap with HIV-related stigma. In the early days of the HIV epidemic in high-income settings, HIV was designated as a “gay disease” and was linked to homosexuality and also to injection drug use [60]. Later, in the context of generalized HIV epidemics such as in southern and eastern sub-Saharan Africa, HIV was associated with several stigmatized behaviors including sexual promiscuity (eg, having multiple sexual partnerships) and sex work [61]. However, much current data on stigma relates explicitly to HIV infection. Although much of what we discuss in this paper has relevance for measurement of both HIV-related and sexual behavior stigma, our focus here is on the neglected area of measurement of stigma related to sexual behavior that can affect MSM and sex workers regardless of HIV status, and thereby impact access to testing, prevention, and treatment services for these groups.

The paper first outlines the goals and approaches of a sexual behavior stigma surveillance effort for which to strive. We then discuss the different population groups from whom relevant data might be collected and discuss the strengths and weaknesses of the potential data collection platforms reaching these groups. Second, we discuss the strengths and weaknesses of current sexual behavior stigma measurement methods among these different groups. We conclude by outlining an agenda for both action and research to strengthen surveillance of sexual behavior stigma.

## A Vision for Sexual Behavior Stigma Surveillance

The primary objective of health surveillance is to (1) monitor trends in health issues for specific populations and (2) characterize the determinants of those most at risk for adverse health outcomes in order to inform public health action [62]. Key components of surveillance systems thus include accurate measurement or diagnostic tools for the phenomenon of interest and the application of epidemiological study designs and analysis that allow comparisons across time and place. The basic components of HIV surveillance for key populations have traditionally focused on monitoring risk practices and coverage of individual level interventions including HIV testing, condom distribution, and treatment [34]. We suggest that this agenda should be expanded to routinely include information on sexual behavior stigma.

In Table 1, we map the major approaches for potential surveillance of sexual behavior stigma. This focuses on 4 population groups from which data could be collected: (1) members of key population groups themselves (regardless of HIV status), who may experience sexual behavior stigma, (2) PLHIV, who are also members of key populations and thus at particularly high risk of experiencing intersecting stigma, (3) members of nonkey population groups whose attitudes and behaviors toward members of key populations are the drivers of the stigma experienced by key populations in communities, and (4) health workers, whose attitudes and behaviors toward members of key populations are the drivers of the stigma experienced by key populations within health settings. In the case of the first two groups, opportunities to capture data on experiences of stigma are feasible through surveys, population-based cohorts, and through service delivery platforms. Relevant data for groups three and four can be collected through surveys and cohort studies of nonkey populations and health workers. Over the next section, we discuss current experiences from each of these approaches, reflecting on potential strengths and weaknesses of each.

**Table 1:** Summary of relevant populations, platforms, and areas of measurement.

Population	Data collection platforms	What can be asked
Key populations (regardless of HIV^a^ status)	Surveys or cohorts using specialized sampling methodology such as RDS^b^, TLS^c^, or Internet	Experienced, perpetrated, perceived, anticipated, and internalized stigma as a result of sexual behavior
	Targeted service delivery platforms contacts	
PLHIV^d^ who are also members of key populations	Representative surveys of PLHIV^d^ that are also able to collect data on stigmatized sexual practices; surveys or cohorts using specialized sampling methodology such as RDS^b^, TLS^c^, or Internet	Experienced, perpetrated, perceived, anticipated, and internalized stigma as a result of sexual behavior or HIV^a^ status
	HIV^a^ treatment and care programs that are also able to collect data on stigmatized sexual practices	
Nonkey populations (regardless of HIV^a^ status)	Population surveys or cohorts	Stigmatizing attitudes toward sexual practice; Perceptions or observations of stigma or discrimination; Report of own discriminatory behaviors
Health workers	Health worker surveys or cohorts	


^a^HIV: human immunodeficiency syndrome.

^b^RDS: respondent-driven sampling.

^c^TLS: time-location sampling.

^d^PLHIV: people living with HIV.

### Data From Key Populations, Regardless of HIV Status

Respondent-driven, time-location, and other hidden-population sampling methods offer survey-based approaches to reaching key population groups and asking about their experiences, perceptions, or anticipations of sexual behavior stigma. These approaches have already been implemented in a variety of settings. For example, in a range of surveys, 20.1% (68/338) of MSM in Malawi reported being afraid to seek health services [63], 46.1% (149/323) reported experiencing stigma in Swaziland [64], 76.2% (170/223) reported experiencing at least one form of discrimination in Lesotho [65], and up to 40% reported experiencing at least one discrimination event in Malawi (34.3%; 68/198), Botswana (56.9%; 66/116), and Namibia (41.5%; 88/212) [66,67]. About one-quarter (25.9%; 117/451) of MSM from a respondent-driven sampling (RDS) study in Vietnam reported high enacted homosexual stigma, 24% (108/451) reported high perceived community stigma, and 28.8% (130/451) reported high internalized homosexual stigma [68]. Also in Vietnam, 12.8% (38/297) of male sex workers reported being hit, beaten, or sexually assaulted for engaging in sex work, and 61.1% (182/298) reported feeling afraid of being harassed or arrested by the police for engaging in sex work [69]. More than 60% of female sex workers responded with agreement or strong agreement to each item of a 10-item sex worker stigma index, which measured perceived stigma from the community and family members [70]. In China, 33.6% (242/721) of surveyed female sex workers reported that AIDS is a punishment for bad behavior, and 36.3% (262/721) agreed that people who get HIV through sex or drug use get what they deserve [71].

More recently, data have suggested a marked similarity in the prevalence of experienced, anticipated, and perceived sexual behavior stigma across settings. Recent analyses from Johns Hopkins and Emory University compared the prevalence of sexual behavior-related stigma toward MSM using data from the US and western and southern sub-Saharan Africa [72]. These data indicated that MSM in the US report similar, and in many cases, higher levels of stigma than MSM in sub-Saharan Africa because of their sexual practices, particularly from family members and health care workers. However, these comparisons are limited because the studies employed different sampling and survey methodologies across settings.

Cohort studies of key populations that measure stigma over time are rarer; although at least a few examples have been documented. The TRUST or RV368 study of MSM in Nigeria measured prevalence of stigma toward MSM before and after passage of the Same Sex Marriage Prohibition Act, demonstrating significantly higher reported occurrences of stigma after the passage of this discriminatory law [24]. Another pilot cohort of MSM in Senegal is currently assessing change in stigma over time as a result of community-based stigma mitigation interventions and MSM sensitization training for health workers [73]. Internalized homophobia has been measured over time in the Multicenter AIDS Cohort Study, an ongoing prospective study of MSM in the United States [74]. In Canada, the Maka Project Partnership includes a community-based cohort of female sex workers that reports on barriers to accessing health services [33,75]. Key population cohort studies lend further evidence for causality between experienced stigma and adverse health effects, and can be used to monitor change in stigma over time. However, these studies are often limited by time, feasibility, and cost constraints.

In addition to survey and cohort studies, another opportunity to ask questions about the experience of stigma might be through HIV testing and other service delivery platforms that reach key populations. However, this approach does not appear to have been widely implemented to date, reflected by a current lack of literature on this topic.

### Data From People Living With HIV Including Those Who Also Self-Report Stigmatized Sexual Behaviors

A hugely important source of data on HIV-related stigma has been the PLHIV stigma index, a survey-based surveillance effort to understand the experiences and trends of stigma toward PLHIV. This PLHIV led and designed tool has been implemented in more than 70 countries, more than 1500 PLHIV have been trained as interviewers, and 85,000 PLHIV have been interviewed [76]. Although focused on PLHIV overall, the results can provide useful information on HIV stigma experienced by key populations living with HIV, in that individuals are asked to disclose membership in a key group such as sex workers, gay, bisexual or other MSM, and people who inject drugs, among others. The standard survey allows for respondents to identify as being a “member” of up to as many as 9 key populations in keeping with the definitions suggested by the UNAIDS terminology guidance [77]. For example, in Belize, 23% of respondents who reported experiences of stigma for reasons other than their HIV status indicated that the stigma they faced was from their lesbian, gay, bisexual, or transgender (LGBT) identity [78]. In the Philippines, those who identified as gay or MSM reported much higher rates of exclusion from religious activities (16.2% to 7%), family activities (13.5% to 9.3%), and of verbal harassment (35.1% to 23.3%), as compared with non-MSM men. In particular, 48.6% of Filipino MSM living with HIV reported suicidal feelings because of their HIV status, a percentage more than 30% higher than the next highest gender group (women at 36.7%) [79]. In a focus group with MSM conducted as part of the Jamaican PLHIV stigma index, high levels of fear about discrimination based on HIV status were expressed, including one respondent’s characterization of disclosure as a “death sentence” [80]. In Vietnam, 6.5% of female sex workers and 2% of MSM reported physical assaults in the last 12 months, compared with 1.5% of women who were not sex workers and 0.9% of non-MSM men. Overall, in the Vietnam study, MSM reported the highest levels of social stigma and self-stigma of any group of PLHIV, followed by female sex workers and people who inject drugs [81].

Beyond violating the human rights of key populations living with HIV, the stigma experiences captured by the PLHIV stigma index can have serious health implications. In Ukraine, for example, sexual behavior stigma appears to increase hesitancy to take an HIV test. The percentage of PLHIV respondents in Ukraine indicating that they feared HIV testing because people may learn about their sexual or drug use behaviors increased from 10% to 18% between 2010 and 2013 [82]. In Philippines, MSM living with HIV were more likely to report that they avoided going to the hospital (27%) or clinic (37.8%) when they needed care than non-MSM men (23.3% and 32.6%) or women (23.3% and 33.3%) [79]. Further, in Malawi, lower percentages of LGBT PLHIV reported taking antiretroviral therapy (ART) or medications to prevent or to treat opportunistic infections than non-LGBT respondents [83].

However, a key challenge to successfully collecting these data through the PLHIV stigma index relies on reaching enough PLHIV survey respondents who are also members of key populations and on ensuring that key population respondents feel comfortable to accurately report their identifications. Apart from the PLHIV stigma index, several other smaller cross-sectional studies have collected behavioral data on MSM and sex workers, including the measurement of HIV status both self-reported and laboratory diagnosed. However, a smaller fraction of these studies have collected stigma information pertaining both to HIV status and sexual behaviors [84,85].

### Data From Other Populations

Most conceptions of stigma hold within them the notion that stigma arises from a separation between those who do and do not carry a stigmatized trait: the “us” and “them,” in short hand. Stigma experienced by key populations therefore results from the actions, beliefs, or perceived beliefs about sexual behavior and key populations held in the communities in which they live. Monitoring these attitudes from other population groups is therefore another important component of sexual behavior stigma surveillance.

A number of tools for this purpose have been developed and applied. For example, one of the most commonly cited scales for measuring stigma toward MSM includes the Attitudes toward Lesbians and Gay Men Scale, which has been used in several studies since 1984 and revised multiple times and most recently in 2004 [86]. Other examples include the Reactions to Homosexuality Scale [87], and the Modern Homonegativity Scale [88]. For sex workers, scales are used less consistently across studies but include the Attitudes Toward Prostitution Scale [89] and the Attitudes Toward Prostitutes and Prostitution Scale [90]. In addition, validated indicators for HIV-related stigma have been developed for monitoring the 2016 United Nations Political Declaration on HIV and AIDS, including negative manifestations of HIV-related stigma, fear of HIV transmission, and discriminatory attitudes toward PLHIV [91].

Validated scales have been used across several settings to determine the attitude toward homosexuality and sex work among specific populations such as students or health workers, or among members of the general population [70,92-95]. An even greater number of studies have measured attitudes toward key populations, but without using validated scales. For example, in a survey of over 1000 university students in Jamaica measuring attitudes toward PLHIV, less than half reported sympathetic attitudes toward MSM (40%) or female sex workers (44%) living with HIV, although 67% and 81% reported sympathetic attitudes toward heterosexual men and nonsex worker women, respectively [96].

A more recent study estimated population-level trends in HIV stigma using data from the Demographic and Health Surveys and AIDS Indicator Surveys of 31 African countries between 2003 and 2013 [97]. These findings pointed to a decline in social distancing from PLHIV, supported by responses to two questions that have been indicated in field tests to be useful for measuring discriminating attitudes [98]. The authors also conclude an increase in anticipated stigma toward PLHIV. However, this conclusion was based on survey questions that have been suggested to be problematic based on cognitive interviewing, in that respondents likely have varying interpretations of these questions. Thus, we caution the interpretation of this trend and emphasize the importance of validating stigma-related survey instruments.

### Data From Health Workers

Data from key population stigma surveys suggest that stigma experienced and perceived in health settings is widespread and acts as a barrier to accessing health services. Many of the same kinds of scales and questions as described above could be applied to health workers as they have been in other nonkey populations. Such work is ongoing, for example, a survey of 332 staff members from health facilities and social service agencies in Jamaica and The Bahamas found that 77% believed that homosexuality is immoral, 72% believed that sex work is immoral, and 51% believed that HIV spreads due to immoral behavior [99]. A brief measurement tool to assess HIV among health care workers validated in 6 countries included questions assessing willingness to provide services to members of various key population groups if the provider had a choice. The study found that on average 13.1% and 12.4% of 1593 surveyed health care workers would prefer not to care for MSM and sex workers, respectively [100].

Another example of implementing stigma measures in a health service setting is the ancillary study of the HPTN071 or PopART trial. In this study, a cluster-randomized trial is being conducted in 21 communities in Zambia and South Africa. The ancillary study enrolled a take-all sample of both facility and community-based health workers, who are involved in the delivery of HIV testing and treatment services across all 21 communities, into a 3-year cohort study running alongside the main trial. Questions included in the cohort surveys include attitudes toward PLHIV, “women who sell sex” and “men who have sex with other men,” as well as perceptions of the way that these groups are treated by coworkers [84].

## Addressing the Challenges to Strengthening Sexual Behavior Stigma Surveillance

As mentioned in the above section, there are clear opportunities to undertake surveillance of HIV-related stigma within surveys, cohort studies, and through service delivery platforms, and much work has already been undertaken. Mainstreaming sexual behavior stigma surveillance to more fully meet the vision described above will require this work to continue and to address directly the challenges and potential biases, which fall broadly into two types: measurement biases and selection biases.

### Measurement Biases

Ongoing surveillance of sexual behavior stigma has the potential to be undermined through three major types of measurement bias. Confronting these is a key challenge that must be addressed. First, the validation of appropriate stigma metrics is needed. As described above, several stigma metrics for MSM and sex workers exist, and careful testing and validation is required for these.

Second, greater harmonization across time and settings in the use of these validated metrics is needed to support comparisons. Stigma takes many forms and is conceptually complex. It is perhaps therefore not surprising that a range of measures have been developed covering overlapping perspectives on stigma. However, in working toward a vision for surveillance of sexual behavior stigma, more widespread adoption of a much smaller number of approaches will be essential to facilitate comparisons over time and place. Although some details on the source or intersection of stigma may be lost by such harmonization, there is a much greater gain to be had in strengthening the capacity to track trends. The current lack of trend data on sexual behavior stigma, as well as HIV stigma, is a direct result of this problem and must urgently be addressed going forward. In addition, there is limited consensus on the appropriate time periods of exposure for stigma, necessitating understanding the acute and chronic effects of exposure to stigma among key populations. Specifically, this includes understanding whether the effects of stigma in potentiating HIV risks among key populations are cumulative, leading to chronic elevated stress levels, or whether acute instances of stigma are more determinative in deciding the likelihood of uptake of health services. Ensuring consistency of measurement similar to other surveillance measures necessitates standardization of data collection instruments and sampling methods.

Third, the potential for social desirability and other reporting biases to bias assessments of prevalence and trends in sexual behavior stigma must be addressed. One method of evaluating the extent of social desirability is by measuring the prevalence of other sensitive topics, such as prevalence of condomless anal sex, to estimate the rate of under- or overreporting of stigma. Another method is by including social desirability scales and testing associations between the scale and variables of interest [101]. However, this remains a relatively poorly understood area. Biased reporting has proved a major barrier to the widespread adoption of sexual behavior surveillance data. On the other hand, methodological research has identified approaches that can improve reporting such as the use of trained and key population-friendly interviewers, Web-based assessments, or the use of audio computer-assisted self-interview tools [102,103].

Other potential measurement biases pertain to misclassification, as well as to the particular phrasing of questions. Exposure misclassification (eg, failure to disclose membership in a key population) can also occur in treatment program data and may be differential with respect to HIV status or other factors. Factors such as age, education level, medical history, internalized homophobia, and connectedness with the LGBT community can affect disclosure of sexual orientation to health care providers, according to a 2012 study of lesbian, gay, or bisexual (LGB) individuals in New York City, which is a city considered to be a “safe space” for gay men and other MSM [104]. In the same study, 90% of gay men disclosed their sexual orientation to a health care provider although only 60% of bisexual men disclosed [104]. Indeed, the likely underreporting of key population membership in health care or HIV treatment program settings would universally challenge surveillance efforts for MSM and sex workers.

Finally, survey questions that ask whether key population participants have ever witnessed a stigmatizing event occur may avoid social desirability bias, but they are problematic in the sense that they are dependent on a particular respondent’s network size. For example, respondents with larger social networks may be more likely to witness or hear about stigmatizing events occurring to the people within their network, as compared with those with smaller networks. Questions that ask about one’s own perpetration of stigma toward key population could be used to avoid this network size bias, but responses to these questions would also be highly susceptible to social desirability biases.

### Selection Biases

The second set of potential biases comprises selection biases that relate to the sampling approaches used to measure stigma toward key populations. Although a full discussion of such biases is beyond the scope of this paper, we note a few generic issues that would be of relevance to sexual behavior stigma. First, research remains ongoing on the representativeness of sampling strategies often used to reach hidden, key populations such as time-location or network-based sampling approaches. Novel approaches such as Web-based surveillance also hold great promise [105]. In all cases, a growing literature describes approaches to operationalizing, quality assurance checking, and analyzing data from such approaches so that the representativeness of the estimates derived from these samples is understood and the uncertainty in estimates is appropriately described. One growing area of interest is extrapolation of information from location-based samples to national-level estimation, and this would be as relevant for stigma metrics as it is for other indicators [106].

Furthermore, there has been debate regarding the appropriate sampling methodology for large surveillance efforts including the PLHIV stigma index, particularly for generating a representative sample across settings. It is presently argued that RDS, a peer-driven chain referral recruitment method, is best practice for sampling of hard-to-reach populations for HIV surveillance [107]. It has also been indicated that RDS can succeed at reaching members of key populations that are less engaged in HIV testing and less likely to be aware of living with HIV [108]. RDS is recommended in settings where time-location or venue-based sampling might overestimate measures of interest such as exposure to HIV prevention services or involvement in the gay community.

Data collected through service delivery platforms have both advantages and disadvantages. Such routine data are often cheap to collect and voluminous. However, the obvious disadvantage is that data are not collected from those who do not access services. This poses a challenge for representativeness and generalizability, but has not undermined the central importance of, for example, data on HIV infection from antenatal-clinic based surveillance or on HIV testing from voluntary counseling and testing clinics. Care and consideration must also be taken to ensure participant safety and sustained access to services, particularly in settings where sexual behaviors are criminalized, or settings with high levels of discriminatory attitudes affecting key populations. Service settings in all contexts should consider establishing plans to learn and respond to these data as a means of optimizing the implementation of their programs. We believe that service delivery platforms offer a potentially huge and important source of information on stigma, and that the utility of these data should be investigated as a matter of urgency.

## Discussion

### Summary

There is a sustained and often increasing burden of HIV in key populations worldwide, with uptake of HIV prevention and treatment services impeded by stigma. As a result, evidence-based stigma reduction interventions may ultimately be the key to overcoming the barriers to coverage and retention in ART and preexposure prophylaxis programs for key populations. However, to effectively measure and evaluate potential stigma mitigation or reduction interventions, consistent and validated stigma metrics for key populations should be integrated as a core component of HIV surveillance systems. To achieve this, there is a need to further validate stigma measures for key populations and attention must be given to the potential biases that might undermine surveillance aims.

Appropriate stigma surveillance measures are required to evaluate change in stigma over time and reduction of adverse HIV-related outcomes through specific mediated and modifiable pathways. Already, several studies have employed sensitization trainings for health care workers to increase competency in meeting the health care needs of key populations in many settings worldwide [52-56]. For example, in South Africa, the Anova Health Institute’s *Health4Men* project aims to institutionalize competence in serving MSM in existing public clinics, which has resulted in over 160 competent sites nationally [56]. Only with validated and consistent stigma surveillance metrics, researchers and policy makers will be able to evaluate programming such as what is described here and to determine the ingredients of successful comprehensive intervention strategies.

Methods for testing the linkage of stigma surveillance data with individual-level biological outcomes among key populations would be needed to provide further evidence for adverse health effects of stigma, as well as potentially provide new perspectives to the surveillance data. There is currently much data to support the notion that stigma increases risk for adverse health outcomes, including mental health, depression, and also risk for HIV via fear or avoidance of health care seeking. However, the majority of studies rely on cross-sectional surveys and evidence would be bolstered through the use of prospective cohort data. Strong, high-quality surveillance data will ultimately be needed to change administrative policies such as the criminalization of sex work or same-sex practices that persist in many countries. However, although legal protections may reduce stigma in some instances, legislative protection alone is not sufficient to eliminate stigma [109]. The first step to reducing the harmful effects of stigma on key populations is the appropriate surveillance and documentation of stigma phenomena.

Another key remaining issue is that there is significant work to be done to characterize intersectionality of HIV-related stigmas. Most studies have focused on internalized stigma, without reconciling the potential nonadditive effects of membership in one or more key populations, as well as HIV status, socioeconomic status, gender identity, and so on [110-113]. Moving forward, it may be useful to compare the strength of association between HIV-status stigma and treatment outcomes with the strength of association between sexual behavior stigma and treatment outcomes, among those living with HIV. Among those not living with HIV, we suggest further assessment of whether anticipated HIV stigma or the various types of sexual behavior stigma (anticipated, internalized, perceived, experienced, and so on) more strongly predict HIV risk. The results of such analyses may be used in future efforts to harmonize these measures for surveillance purposes. Although quantifying the intersectionality of these stigmas may allow for a more robust HIV response, there may be challenges to selecting the fewest number of survey items to accurately assess intersectionality while also attempting to reduce participant burden. It remains unclear whether it is important to collect the reason or attribute (eg, sexual practices, sexual or gender identities, occupation, socioeconomic status) thought to be causing the increased stigma or whether it sufficient to characterize the nonadditive burden of stigma [114].

### Recommendations

Once the best practices of stigma surveillance have been established, monitoring trends over time will be important for many reasons. These include measuring the burden of stigma; guiding the planning, implementation, and evaluation of programs to prevent and mitigate stigma; detecting changes in public opinion or behavior; anticipating future trends; and providing a basis for epidemiologic investigation. Because very limited longitudinal data exist characterizing stigma affecting key populations, there are limited opportunities to assess the causality of stigma and its potential health effects, which is necessary for filling data gaps and motivating intervention resources. Thus, moving forward necessitates the integration of validated stigma scales during HIV epidemiologic, surveillance, and intervention studies focused on key populations to inform and establish comprehensive HIV and general health programming.

## Abbreviations

AIDS: acquired immunodeficiency syndrome
ART: antiretroviral therapy
HIV: human immunodeficiency virus
LGB: lesbian, gay, or bisexual
LGBT: lesbian, gay, bisexual, or transgender
MSM: men who have sex with men
PLHIV: people living with HIV
RDS: respondent-driven sampling
TLS: time-location sampling
UNAIDS: Joint United Nations Program on HIV and AIDS
